# Trimetazidine attenuates dexamethasone-induced muscle atrophy via inhibiting NLRP3/GSDMD pathway-mediated pyroptosis

**DOI:** 10.1038/s41420-021-00648-0

**Published:** 2021-09-18

**Authors:** Li Wang, Xin-Feng Jiao, Cheng Wu, Xiao-Qing Li, Hui-Xian Sun, Xi-Yu Shen, Kang-Zhen Zhang, Can Zhao, Li Liu, Man Wang, Yun-Ling Bu, Jia-Wen Li, Fan Xu, Chen-Lu Chang, Xiang Lu, Wei Gao

**Affiliations:** 1 Department of Geriatrics, Sir Run Run Hospital, Nanjing Medical University, Nanjing, China; 2 Key Laboratory for Aging & Disease, Nanjing Medical University, Nanjing, China; 3 Department of Geriatrics, The Second Affiliated Hospital of Nanjing Medical University, Nanjing, China

**Keywords:** Musculoskeletal abnormalities, Skeletal muscle

## Abstract

Skeletal muscle atrophy is one of the major side effects of high dose or sustained usage of glucocorticoids. Pyroptosis is a novel form of pro-inflammatory programmed cell death that may contribute to skeletal muscle injury. Trimetazidine, a well-known anti-anginal agent, can improve skeletal muscle performance both in humans and mice. We here showed that dexamethasone-induced atrophy, as evidenced by the increase of muscle atrophy F-box (Atrogin-1) and muscle ring finger 1 (MuRF1) expression, and the decrease of myotube diameter in C2C12 myotubes. Dexamethasone also induced pyroptosis, indicated by upregulated pyroptosis-related protein NLR family pyrin domain containing 3 (NLRP3), Caspase-1, and gasdermin-D (GSDMD). Knockdown of NLRP3 or GSDMD attenuated dexamethasone-induced myotube pyroptosis and atrophy. Trimetazidine treatment ameliorated dexamethasone-induced muscle pyroptosis and atrophy both in vivo and in vitro. Activation of NLRP3 using LPS and ATP not only increased the cleavage and activation of Caspase-1 and GSDMD, but also increased the expression levels of atrophy markers MuRF1 and Atrogin-1 in trimetazidine-treated C2C12 myotubes. Mechanically, dexamethasone inhibited the phosphorylation of PI3K/AKT/FoxO3a, which could be attenuated by trimetazidine. Conversely, co-treatment with a PI3K/AKT inhibitor, picropodophyllin, remarkably increased the expression of NLRP3 and reversed the protective effects of trimetazidine against dexamethasone-induced C2C12 myotube pyroptosis and atrophy. Taken together, our study suggests that NLRP3/GSDMD-mediated pyroptosis might be a novel mechanism for dexamethasone-induced skeletal muscle atrophy. Trimetazidine might be developed as a potential therapeutic agent for the treatment of dexamethasone-induced muscle atrophy.

## Introduction

The loss of muscle mass and strength or physical function (sarcopenia) is one of the common disabilities in the elderly [1]. It occurs under various conditions, such as aging [2], cancer [3], cardiovascular diseases [4], and drug treatment [5]. Glucocorticoids are known to have catabolic effects on skeletal muscle, either as an endogenous endocrine hormone released in response to various stressful conditions or as a drug given exogenously to treat inflammation [6]. However, the sustained elevated levels of glucocorticoids result in a decrease in protein synthesis and an increase in proteolysis in skeletal muscle, ultimately leading to muscle atrophy and frailty [7, 8]. Although several growth factors have been indicated that may play important roles in mediating glucocorticoids’ effects on muscle mass and function [9], the exact underlying molecular mechanisms remain unclear and the effective treatment for glucocorticoid-induced muscle atrophy needs to be further elucidated.

Despite the extensive use of glucocorticoids as anti-inflammatory agents, emerging evidence indicates that glucocorticoids can also activate inflammatory signaling pathways as a mechanism for inducing muscle atrophy [10]. Pyroptosis is a novel pro-inflammatory programmed cell death, which is characterized by the cleavage of the pore-forming protein gasdermin-D (GSDMD) and the formation of cytotoxic pores in the plasma membrane [11]. To date, the best characterized upstream activators of pyroptosis are inflammasome-signaling pathway components, such as nucleotide oligomerization domain (NOD)-like receptors (NLRs) [12]. Activation of NLRP3 inflammasome triggers Caspase-1-dependent pyroptosis accompanied by the release of inflammatory factors, such as interleukin (IL)−1β and IL-18 [13]. Recent studies have demonstrated that pyroptosis may play an important role in the pathogenesis of muscle loss and weakness [14]. However, the mechanism to which pyroptosis contributes to the atrophy of skeletal muscle induced by dexamethasone remains unclear.

Trimetazidine has been used as an anti-anginal agent for decades. Trimetazidine exerts cardioprotective effects by shifting energy production from fatty acid oxidation to glucose oxidation [15]. Besides its cardiac protective ability, recent evidence suggests that trimetazidine can also improve skeletal muscle performance both in humans and mice [16, 17]. Molinari F et al. [18] showed that trimetazidine could act like an ‘exercise mimetic’ in cancer cachexia through increasing protein synthesis and reducing protein degradation. Another study found that trimetazidine protected muscle cells against starvation or inflammation-induced atrophy by inhibiting protein degradation and inducing autophagy [19]. In the statin-induced skeletal muscle injury model, trimetazidine could alleviate simvastatin-induced exercise intolerance and muscle damages by ameliorating energy metabolism dysfunction [20]. However, studies examining the benefits of trimetazidine in glucocorticoid-induced skeletal muscle atrophy are limited. Interestingly, trimetazidine has recently been shown to ameliorate lipopolysaccharide (LPS)-induced cardiomyocyte pyroptosis by promoting neutrophil migration to cardiac tissue [21]. In the present study, we sought to explore the role and underlying mechanism of trimetazidine in the regulation of pyroptosis in a dexamethasone-induced muscle atrophy model.

## Materials and methods

### C2C12 myoblasts culture and differentiation

Murine C2C12 myoblasts were obtained from ATCC and incubated at 37 °C, 5% CO_2_ in DMEM with 80U/ml penicillin and 0.08 mg/ml streptomycin and 10% fetal bovine serum (Gibco). For the induction of differentiation into myotubes, sub-confluent myoblasts were switched to DMEM containing 2% horse serum (Biological Industries, Israel), and then cultured for 4 days. The myotubes were treated with 10 μM dexamethasone for 24 h [46], and 150 μM trimetazidine was added in the last 6 h [19]. To activate NLRP3, after treated with 10 μM DEX and/or 150 μM TMZ, C2C12 myotubes were cultured in a medium containing 100 ng/mL LPS (Sigma-Aldrich, US) for 2 h, followed by adding 2.5 mM ATP (Solarbio, China) and cultured for another 1 h [24]. For inhibiting phosphoinositide 3-kinase (PI3K)/AKT pathway, C2C12 myotubes were cultured in a medium containing 2.5 μM picropodophyllin (PPP) (MCE, China) for 24 h [24].

### Cell viability

C2C12 cells were seeded in 96-well plates at a density of 1 × 10^4^ cells per well. Myotubes were treated with incremental dosages of dexamethasone (0.1, 1, 10 μM) for 24 h, and different concentrations of trimetazidine (50, 100, 150, 200 μM) were added in the last 6 h. CCK-8 reagent (Dojindo, Japan) was added to each well and the absorbance was measured at 450 nm after 2 h incubation by a microplate reader (Synergy H1, BioTek, US). The relative cell viability was calculated according to the instructions.

### Small interfering RNA (siRNA)

Chemically synthesized double-stranded siRNA duplexes targeting mouse NLRP3 (No. siG1161091244) and GSDMD (No. siG180420040947) were obtained from RiboBio Co., Ltd. (Guangzhou, China). C2C12 myotubes were transfected with 50 nM siRNAs at day-4 post-differentiation by using RNAiMAX (Invitrogen, US) for 24 h following treatment with dexamethasone.

### Mice

C57BL/6 J male mice of 8 weeks old were housed at a controlled temperature of 24 ± 2 °C and relative humidity of 45 ± 15% with a 12 h light/dark cycle. Mice were randomized into four groups (*n* = 8) and intraperitoneally injected accordingly for 10 days: control (0.9% saline), dexamethasone (25 mg/kg) (D4902, Sigma) [46], trimetazidine (5 mg/kg) (653322, Sigma) [17], dexamethasone (25 mg/kg)+trimetazidine (5 mg/kg). All of the animal experiments were carried out under the approval of the Animal Ethics Committee of Nanjing University and complied with the National Institutes of Health guide for the care and use of laboratory animals (NIH Publications No. 8023, revised 1978)

### Running test

An exercise tolerance test was performed at the end of the experiment using a mouse treadmill (Zhishuduobao, DB030, China). The running protocol referred to previously published articles [20]. The time and distance were recorded when the mice were exhausted. The criterion for exhaustion was defined as touching the electric grid for more than 5 s [47].

### Grip strength test

A grip strength test was performed at the end of the experiment using a grip strength metre (Softmaze, Shanghai, China). Briefly, to assess forelimb strength, mice were allowed to rest on a T-bar such that they could tightly grip the T-bar using only the two forelimbs. The tail of each mouse was pulled directly toward the tester and parallel to the T-bar with the same force. Grip strength was calculated according to the instrument software instructions.

### Protein extraction and Western blot analysis

Myotubes or gastrocnemius samples were lysed with cold RIPA buffer (Beyotime Biotechnology, Shanghai, China) containing 1 mM NaF, 1 mM sodium orthovanadate, and 1 mM phenylmethylsulfonyl fluoride. An equal amount of protein was separated by 4-20% SDS-PAGE (GenScript Biotechnology, Nanjing, China), transferred to PVDF membranes (Millipore, Billerica, MA, USA), and then blocked with 5% nonfat milk. Membranes were incubated with specific primary antibodies overnight at 4 °C. The horseradish peroxidase-conjugated secondary antibody was incubated for 1.5 h, then the immune complexes were detected by Immobilon Western HRP Substrate Peroxide Solution (Millipore Corporation, Billerica, MA 01821, USA). Images were acquired using ChemiDocTM XRS + Imaging System (Bio-Rad, USA). Band densitometry measurements were assessed using Image Lab 6.0 software. All the primary antibodies were listed in Table S1.

### Isolation of total RNA and Real-time PCR analysis

Total RNA from C2C12 myotubes samples was isolated by Total RNA Extraction Kit (dp419, TIANGEN, China). cDNA was synthesized using PrimeScript™ RT reagent Kit (Perfect Real Time) (RR047A, Takara, Japan). Quantitative Real-time PCR was performed using Maxima SYBR Green/ROX qPCR Master Mix (2X) (Thermo Scientific, K0221, USA) on QuantStudio 5 system (Applied Biosystems, USA). The relative gene expression levels were calculated by the 2^−ΔΔCt^ method using Glyceraldehyde 3-phosphate dehydrogenase (GAPDH) as an internal control. All the primer sequences were listed in Table S2.

### Immunofluorescence and myotube diameter measurement

C2C12 cells were fixed with 4% paraformaldehyde for 20 min, permeabilized with 0.5% Triton X-100 for 15 min, and then blocked with 5% BSA for 1 h. Cells were incubated with anti-MHC (1:200, MF20, DSHB) overnight at 4 °C, then incubated with secondary antibody Cy3-AffiniPure Rabbit Anti-Mouse IgG (H + L) (1:500, Jackson). Nuclear counterstaining was performed with DAPI. Images were captured using a fluorescence microscope (Zeiss Axio Scope, Germany). Myotube diameter analysis was performed as previously published [48].

### Haematoxylin-eosin (HE) staining

Fresh gastrocnemius samples were fixed with 4% paraformaldehyde overnight and embedded with paraffin, serially sliced to 4 µm for HE staining. For muscle fiber cross-sectional area analysis, images were captured using a microscope (Zeiss Axio Scope.A1, Germany) and calculated by using the ImageJ software (National Institutes of Health, USA) in five random fields of each section.

### Statistical analysis

Data were presented as mean ± SEM and analyzed by SPSS 21.0. Normality of distribution was assessed using the Kolmogorov-Smirnov test. Comparison between two groups was performed with Student’s *t*-tests or Mann–Whitney U tests. For comparison between more than two groups, one-way ANOVA or the Kruskal–Wallis test was used as appropriate. Significance was accepted as *P* < 0.05.

## Results

### Pyroptosis is activated in dexamethasone-induced C2C12 myotube atrophy

**Fig. 1:**
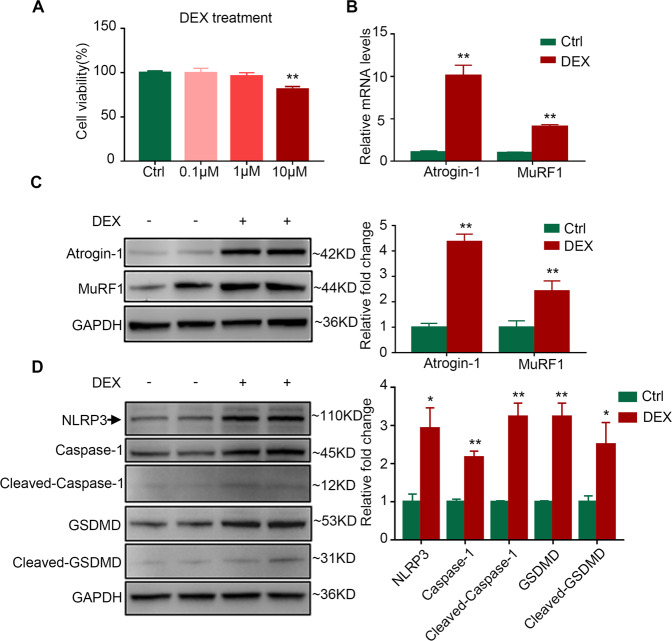
DEX induces muscle atrophy and pyroptosis in C2C12 myotubes. **A** Cell viability of C2C12 myotubes treated with DEX (0.1, 1, 10 μM) for 24 h. *n* = 5 per group. **B** Real-time PCR analysis of expression of muscle atrophic markers (Atrogin-1 and MuRF1) in C2C12 myotubes treated with 10 μM DEX for 24 h. *n* = 6 per group. **C** Western blot analysis of expression level of Atrogin-1 and MuRF1 in C2C12 myotubes treated with 10 μM DEX for 24 h. GAPDH was used as a loading control. *n* = 5 per group. **D** Western blot analysis of expression level of NLRP3, Caspase-1, Cleaved-Caspase-1, GSDMD, and Cleaved-GSDMD in C2C12 myotubes treated with 10 μM DEX for 24 h. *n* = 4 per group. All experiments were performed on C2C12 mature myotubes. Data are presented as mean ± SEM. *P < 0.05 vs. Ctrl, **P < 0.01 vs. Ctrl. DEX dexamethasone, Ctrl control.

Inflammation plays a crucial role in the development of muscle atrophy [13], we first determined whether pyroptosis was activated in dexamethasone-induced myotube atrophy. To form mature myotubes, C2C12 myoblasts were incubated with a differentiation medium for 4 days until cell fusion (Figure S1). Dexamethasone at concentrations of 0.1 and 1 μM did not affect cell viability, while 10 μM dexamethasone induced cell death (Fig. 1A). Moreover, 10 μM dexamethasone significantly increased the mRNA levels of Atrogin-1 and MuRF1 in C2C12 myotubes (Fig. 1B). Similar results were observed in the protein levels of Atrogin-1 and MuRF1 at the dose of 10 μM (Fig. 1C). Importantly, the protein levels of pyroptosis-related molecules, including NLRP3, Caspase-1, Cleaved-Caspase-1, GSDMD, and Cleaved-GSDMD were elevated in dexamethasone-treated C2C12 myotubes (Fig. 1D), indicating that pyroptosis was activated in dexamethasone-induced myotube atrophy.

### Knockdown of NLRP3/GSDMD alleviates dexamethasone-induced myotube pyroptosis and atrophy

**Fig. 2:**
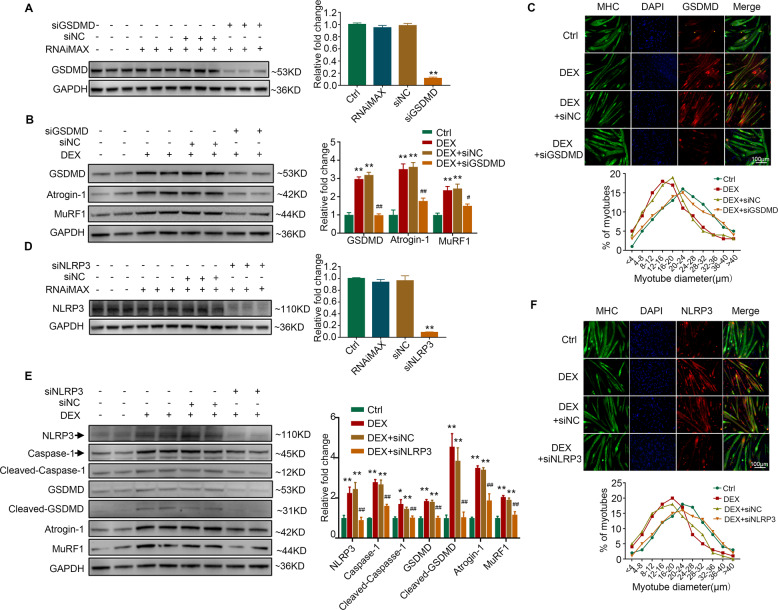
Inhibition of pyroptosis alleviates DEX-induced muscle atrophy in C2C12 myotubes. **A** C2C12 myotubes were transfected with negative control (siNC) or siRNA targeting GSDMD (siGSDMD). Protein levels of GSDMD were analyzed. *n* = 3 per group. **P < 0.01 vs. siNC. **B**–**C** C2C12 myotubes were treated with 10 μM DEX combined with siNC or siGSDMD for 24 h. **B** Protein levels of GSDMD, Atrogin-1, and MuRF1 were analyzed. (**C**) Immunofluorescence staining showed the co-localization of GSDMD (red) with MHC (green) in myotubes. The distribution of C2C12 myotubes diameter was analyzed. *n* = 4 per group. **P < 0.01 vs. Ctrl. ^#^*P* < 0.05 vs. DEX + siNC, ^##^*P* < 0.01 vs. DEX + siNC. Scale bar = 100 μm. **D** C2C12 myotubes were transfected with negative control (siNC) or siRNA targeting NLRP3 (siNLRP3). Protein levels of NLRP3 were analyzed. *n* = 3 per group. **P < 0.01 vs. siNC. (E-F) C2C12 myotubes were treated with 10 μM DEX combined with siNC or siNLRP3 for 24 h. **E** Protein levels of NLRP3, Caspase-1, Cleaved-Caspase-1, GSDMD, Cleaved-GSDMD, Atrogin-1, and MuRF1. **F** Immunofluorescence staining showed the co-localization of NLRP3 (red) with MHC (green) in myotubes. *n* = 4 per group. **P < 0.01 vs. Ctrl. ^#^*P* < 0.05 vs. DEX + siNC, ^##^*P* < 0.01 vs. DEX + siNC. Scale bar = 100 μm. NC normal control, DEX dexamethasone.

Since NLRP3 inflammasome plays a crucial role in the activation of GSDMD-dependent pyroptosis [12], we therefore investigated whether NLRP3/GSDMD pathway was involved in dexamethasone-induced myotube atrophy. We found that dexamethasone-induced increase of Atrogin-1 and MuRF1 expression was alleviated by silencing GSDMD (Fig. 2A, B). Moreover, dexamethasone-induced myotube atrophy was also attenuated by GSDMD inhibition, as evidenced by the percentage of myotubes of larger diameter (Fig. 2C) and increased C2C12 myotube diameter (Figure S2A). In addition, knockdown of NLRP3 (Fig. 2D) abolished the activation of Caspase-1-dependent pyroptosis as well as the increase of Atrogin-1 and MuRF1 induced by dexamethasone (Fig. 2E). The decrease of C2C12 myotube diameter caused by dexamethasone was also attenuated by the silence of NLRP3 (Fig. 2F and Fig. S2B). These results demonstrate that inhibition of NLRP3/GSDMD pathway-mediated pyroptosis could ameliorate dexamethasone-induced myotube atrophy.

### Trimetazidine attenuates dexamethasone-induced C2C12 myotube atrophy

**Fig. 3:**
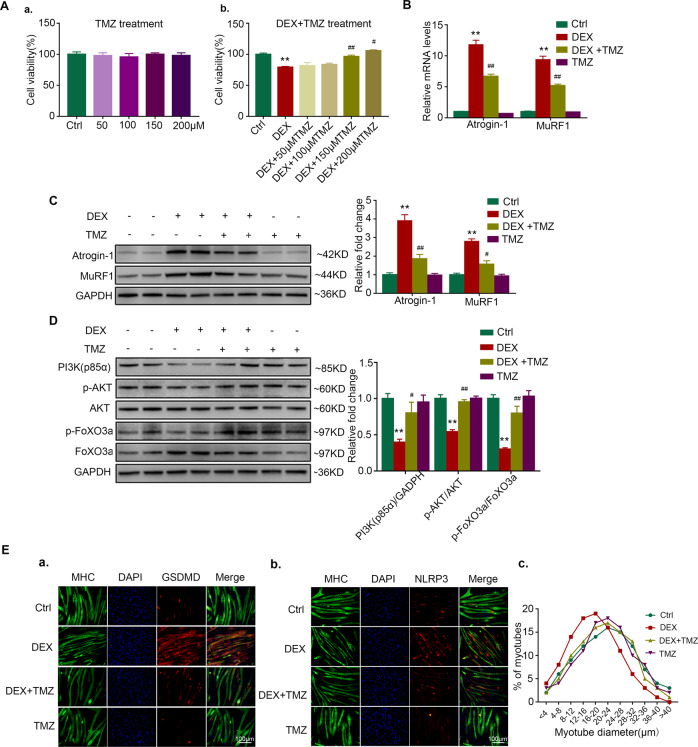
TMZ alleviates DEX-induced muscle atrophy in C2C12 myotubes. **A** Myotubes viability. a. C2C12 myotubes treated with TMZ (50, 100, 150, 200 μM) for 6 h. b. C2C12 myotubes treated with 10 μM DEX for 24 h and TMZ (50, 100, 150, 200 μM) in the last 6 h. *n* = 4–5 per group. **B** Real-time PCR analysis of expression of Atrogin-1 and MuRF1 in C2C12 myotubes treated with 10 μM DEX and 150 μM TMZ. *n* = 4 per group. **C** Protein levels of Atrogin-1 and MuRF1 in C2C12 myotubes treated with 10 μM DEX and 150 μM TMZ. *n* = 4 per group. **D** Protein levels of p85α PI3K, p-AKT, total AKT, p-FOXO3a, and total FOXO3a in C2C12 myotubes treated with 10 μM DEX and 150 μM TMZ. *n* = 4 per group. **E** Immunofluorescence staining for NLRP3 and GSDMD (red) in MHC (green) positive C2C12 myotubes treated with 10 μM DEX and 150 μM TMZ. The distribution of C2C12 myotubes diameter was analyzed. *n* = 4 per group. Scale bar; = 100 μm. **P < 0.01 vs. Ctrl. ^#^*P* < 0.05 vs. DEX. ^##^*P* < 0.01 vs. DEX. DEX dexamethasone; TMZ trimetazidine, Ctrl control.

We further examined the effects of trimetazidine on myotube atrophy in vitro. Adding trimetazidine at concentrations of 50, 100, 150, and 200 μM to C2C12 myotubes did not reduce cell viability (Fig. 3Aa). At the concentration of 150 and 200 μM, trimetazidine protected against dexamethasone-induced cell death (Fig. 3Ab). Adding 150 μM trimetazidine ameliorated dexamethasone-induced upregulation of Atrogin-1 and MuRF1 mRNA levels (Fig. 3B). Similar results were observed in the protein levels of Atrogin-1 and MuRF1 (Fig. 3C). The inhibition of the PI3K/AKT signaling pathway has been implicated in the induction of muscle atrophy by dephosphorylating FoxO3a, which in turn promotes the transcriptional activation of atrogin-1 and MuRF1 [22, 23]. We found that the phosphorylation of PI3K/AKT/FoxO3a was decreased by dexamethasone and were reversed by trimetazidine in C2C12 myotubes (Fig. 3D). Moreover, a dexamethasone-induced decrease of C2C12 myotubes diameters was markedly reversed by trimetazidine (Fig. 3E and Fig. S2C). The results indicate that trimetazidine can also attenuate dexamethasone-induced muscle atrophy in vitro.

### Trimetazidine alleviates dexamethasone-induced muscle atrophy in mice

**Fig. 4:**
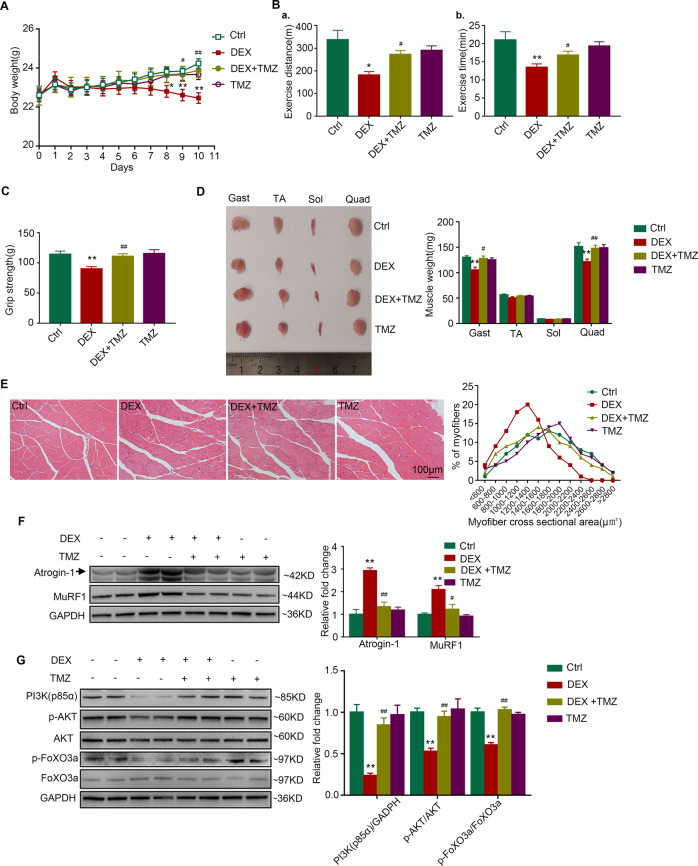
TMZ ameliorates DEX-induced muscle atrophy in mice. Mice were intraperitoneally injected with 0.9% saline (Ctrl), DEX (25 mg/kg), TMZ (5 mg/kg), or DEX (25 mg/kg) +TMZ (5 mg/kg) for 10 days. **A** Body weight of mice. *n* = 8 per group. **B** Exercise capacity testing. a. Running distance. b. Running time. *n* = 8 per group. **C** Grip strength test. *n* = 8 per group. **D** Comparison of representative samples of dissected skeletal muscle including gastrocnemius (Gast), tibialis anterior muscle (TA), soleus muscle (Sol), and quadriceps (Quad). *n* = 4 per group. **E** Representative H&E staining of myofiber cross-section of Gast. Scale bar = 100 μm. A microscope with a 10× objective was used to capture the images. *n* = 4 per group. **F** Protein levels of Atrogin-1 and MuRF1 in Gast muscle. *n* = 4 per group. **G** Protein levels of p85α PI3K, p-AKT, total AKT, p-FOXO3a, and total FOXO3a in Gast muscle. *n* = 4 per group. *P < 0.05 vs. Ctrl. **P < 0.01 vs. Ctrl. ^#^*P* < 0.05 vs. DEX. ^##^*P* < 0.01 vs. DEX. DEX dexamethasone, TMZ trimetazidine, Ctrl control.

To determine the protective effect of trimetazidine against muscle wasting, we established a model of dexamethasone-induced muscle atrophy in mice. The body weight was decreased in mice injected with dexamethasone when compared with control mice (Fig. 4A). The results of the running test indicated impaired exercise capacity in dexamethasone-treated mice, showing decreased running distance (181.94 ± 14.94 vs. 337.71 ± 41.98 m, Figure 4Ba) and running time (13.48 ± 0.94 vs. 21.03 ± 2.28 min, Figure 4Bb). In addition, a decrease in the grip strength (Fig. 4C) of dexamethasone-treated mice was also observed. As expected, trimetazidine alleviated dexamethasone-induced loss of body weight (Fig. 4A), as well as the decline of running distance (273.00 ± 16.88 vs. 181.94 ± 14.94 m, Figure 4Ba), running time (16.84 ± 1.09 vs. 13.48 ± 0.94 min, Figure 4Bb) and grip strength (114.25 ± 5.25 vs. 90 ± 3.79 g, Fig. 4C). Dexamethasone injection also induced significant muscle atrophy, especially in gastrocnemius and quadriceps (Fig. 4D and Figure S3), reduced the myofiber cross-sectional area (Fig. 4E and Fig. S4), and increased the protein expression of Atrogin-1 and MuRF1 (Fig. 4F), whereas trimetazidine treatment attenuated the effects of dexamethasone. Moreover, dexamethasone decreased the expression of p85α PI3K and the phosphorylated levels of AKT and FoxO3a, which was reversed by trimetazidine treatment (Fig. 4G). These data reveal that trimetazidine could attenuate dexamethasone-induced exercise intolerance and muscle atrophy.

### Trimetazidine mitigates dexamethasone-induced pyroptosis in vitro and in vivo

**Fig. 5:**
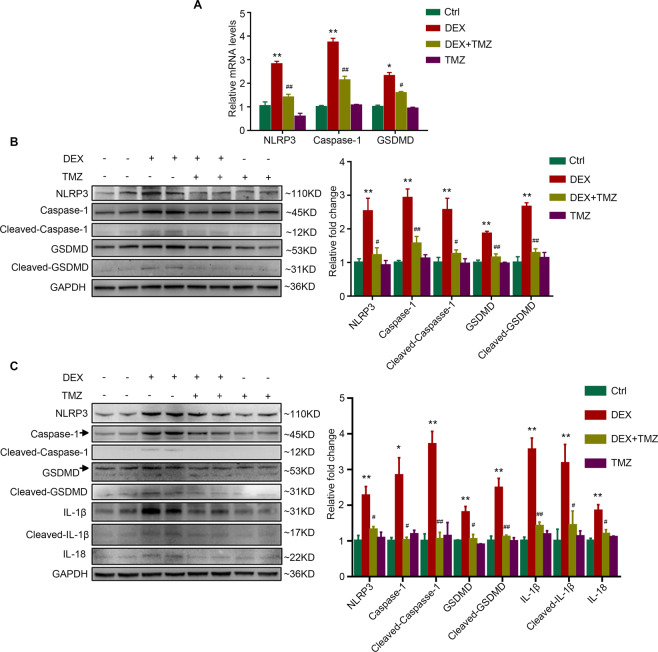
TMZ attenuates DEX-induced pyroptosis in myotubes and mice. **A**–**B** C2C12 myotubes were treated with 10 μM DEX for 24 h combined with or without 150 μM TMZ in the last 6 h. **A** mRNA levels of NLRP3, Caspase-1, and GSDMD. **B** Protein levels of NLRP3, Caspase-1, Cleaved-Caspase-1, GSDMD, and Cleaved-GSDMD. *n* = 4 per group. **C** Mice were intraperitoneally injected with 0.9% saline (Ctrl), DEX (25 mg/kg), TMZ (5 mg/kg), or DEX (25 mg/kg) +TMZ (5 mg/kg) for 10 days. Protein levels of NLRP3, Caspase-1, Cleaved-Caspase-1, GSDMD, Cleaved-GSDMD, IL-1β, Cleaved-IL-1β, and IL-18 were analyzed in gastrocnemius. *n* = 4 per group. *P < 0.05 vs. Ctrl. **P < 0.01 vs. Ctrl. ^#^*P* < 0.05 vs. DEX. ^##^*P* < 0.01 vs. DEX. DEX dexamethasone, TMZ trimetazidine, Ctrl control.

Pyroptosis plays an important role in the development of muscle atrophy [13], and trimetazidine has been shown to have the ability of inhibiting pyroptosis [21]. Treating C2C12 myotubes with dexamethasone increased the mRNA expression of pyroptosis-related molecules, including NLRP3, Caspase-1, and GSDMD (Fig. 5A). Consistently, the protein levels of NLRP3, Caspase-1, Cleaved-Caspase-1, GSDMD, and Cleaved-GSDMD were also elevated in dexamethasone-treated C2C12 myotubes (Fig. 5B). Importantly, the upregulated pyroptosis-related genes induced by dexamethasone were mitigated by trimetazidine (Fig. 5A,B). Similar to the results in C2C12 myotubes, trimetazidine also markedly attenuated the dexamethasone-induced increase of NLRP3, Cleaved-Caspase-1, GSDMD, Cleaved-GSDMD, IL-1β, and IL-18 expression (Fig. 5C) in mice. These results indicate that trimetazidine can protect against dexamethasone-induced pyroptosis in skeletal muscle.

### The protective effect of trimetazidine against dexamethasone-induced C2C12 myotube pyroptosis and atrophy was diminished by activating NLRP3

**Fig. 6:**
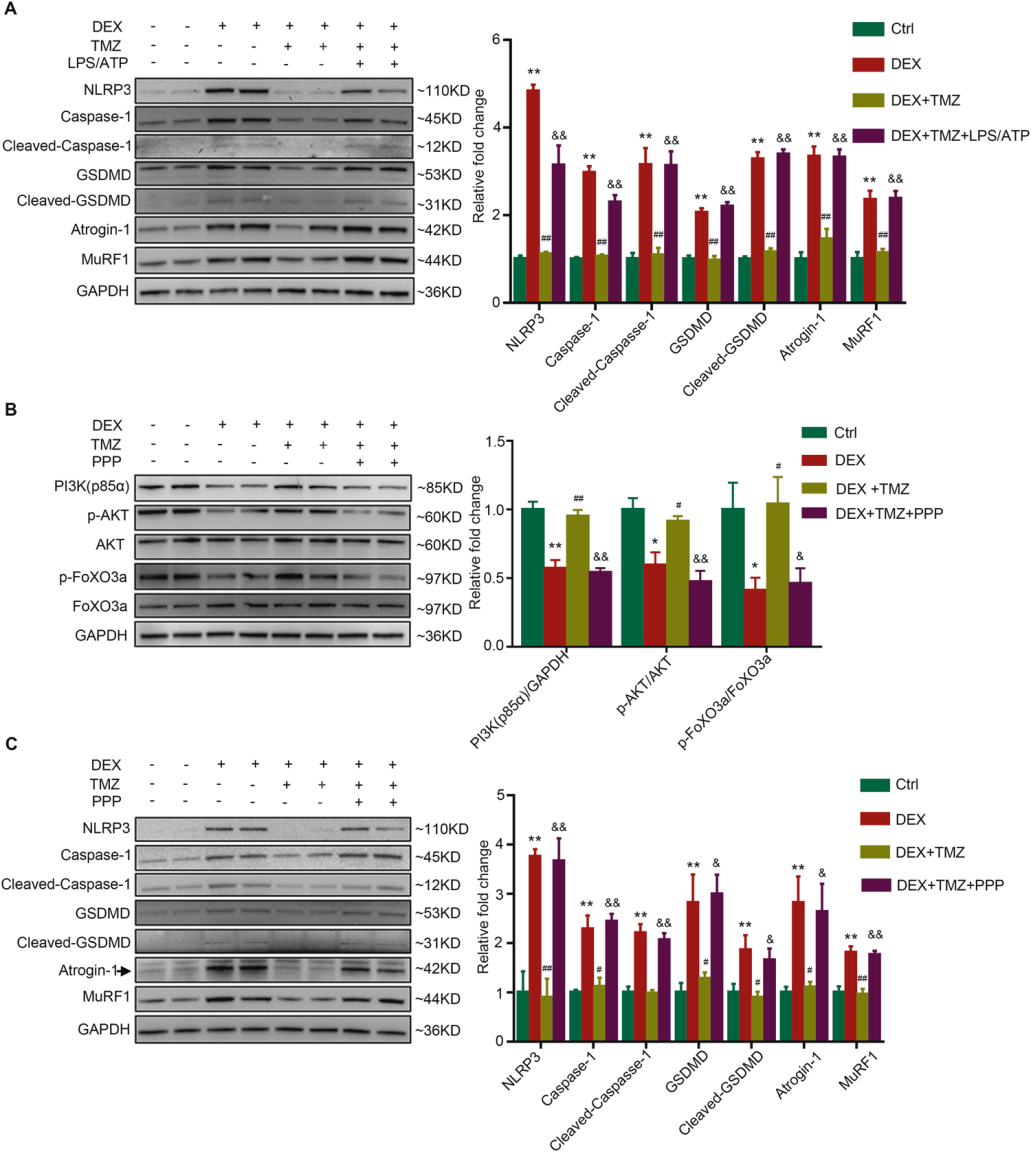
PI3K/AKT pathway is involved in the protective effect of TMZ against DEX-induced pyroptosis and atrophy in C2C12 myotubes. C2C12 myotubes were treated with 10 μM DEX for 24 h combined with or without 150 μM TMZ in the last 6 h. **A** Myotubes were further treated with 100 ng/ml LPS for 2 h and 2.5 mM ATP for 1 h. Protein levels of NLRP3, Caspase-1, Cleaved-Caspase-1, GSDMD, Cleaved-GSDMD, Atrogin-1, and MuRF1 were analyzed. *n* = 4 per group. **B**–**C** Myotubes were further treated with 2.5 μM PPP for 24 h. **B** Protein levels of p85α PI3K, p-AKT, total AKT, p-FoxO3a, and total FoxO3a. **C** Protein levels of NLRP3, Caspase-1, Cleaved-Caspase-1, GSDMD, Cleaved-GSDMD, Atrogin-1, and MuRF1. *n* = 4 per group. *P < 0.05 vs. Ctrl. **P < 0.01 vs. Ctrl. ^#^*P* < 0.05 vs. DEX. ^##^*P* < 0.01 vs. DEX. ^&^*P* < 0.05 vs. DEX + TMZ. ^&&^*P* < 0.01 vs. DEX + TMZ. DEX dexamethasone, TMZ, trimetazidine, Ctrl control, PPP Picropodophyllin.

To investigate whether the suppression of NLRP3 is crucial for the protective effects of trimetazidine in dexamethasone-treated C2C12 myotubes, we further treated C2C12 myotubes with LPS and ATP to activate NLRP3 [24]. As shown in Fig. 6A, the inhibition of dexamethasone-induced cleavage and activation of Caspase-1 and GSDMD by trimetazidine was diminished by LPS/ATP treatment. Moreover, LPS/ATP treatment also resulted in increased expression of atrophy markers, Atrogin-1 and MuRF1, indicating that trimetazidine may attenuate dexamethasone-induced muscle atrophy via inhibiting NLRP3-mediated pyroptosis.

### The protective effect of trimetazidine against dexamethasone C2C12 myotube pyroptosis and atrophy was diminished by inhibiting PI3K/AKT pathway

To verify whether the protective effects of trimetazidine against dexamethasone-induced myotube pyroptosis and atrophy were mediated through activating PI3K/AKT pathway, C2C12 myotubes were treated with a PI3K/AKT pathway inhibitor, picropodophyllin (PPP), together with trimetazidine. When co-treated with PPP, the phosphorylation of PI3K, AKT, and FoxO3a in trimetazidine-treated myotubes were almost abolished (Fig. 6B). Furthermore, co-treatment of PPP diminished the inhibitive effects of trimetazidine on NLRP3, cleavage of Caspase-1, and GSDMD in dexamethasone-treated C2C12 myotubes (Fig. 6C). As expected, PPP treatment almost blocked the effects of trimetazidine on the inhibition of dexamethasone-induced upregulation of Atrogin-1 and MuRF1 (Fig. 6C). Therefore, these data suggest that trimetazidine mitigated dexamethasone-induced pyroptosis and atrophy in C2C12 myotubes via activating PI3K/AKT pathway.

## Discussion

Skeletal muscle atrophy is one of the major side effects of high dose or sustained usage of glucocorticoids [9]. Pyroptosis is involved in the development of muscle atrophy [13]. We here show that dexamethasone treatment induces muscle atrophy and pyroptosis both in vitro and in vivo. Inhibiting pyroptosis by knockdown NLRP3/GSDMD pathway alleviates dexamethasone-induced myotube atrophy. Trimetazidine exerts protective effects on improving dexamethasone-induced skeletal dysfunction in vivo. Such protection relies on the suppression of pyroptosis induced by dexamethasone, since trimetazidine treatment reverses dexamethasone-induced activation of pyroptosis both in C2C12 myotubes and in mice. Therefore, trimetazidine might be a potential therapeutic agent for the treatment of glucocorticoid-induced skeletal muscle atrophy.

Skeletal muscle comprises approximately 40% of body mass and is a major target of glucocorticoids [25, 26]. Plenty of evidence indicates that glucocorticoids can cause muscle atrophy via decreasing protein synthesis and increasing degradation; in particular, the ubiquitin‐proteasome system is the most prominent mechanism of protein breakdown [25, 27]. In the ubiquitin-proteasome pathway, FoxO3a is considered as a key player in the control of skeletal muscle protein turnover [28, 29]. Inhibition of the PI3K/AKT signaling pathway has been implicated in the induction of muscle atrophy via dephosphorylating FoxO3a and promoting the nuclear translocation of FoxO3a, which in turn increases the expression of the E3 ubiquitin ligases, Atrogin-1 and MuRF1 [22, 23]. As expected, our data showed that the phosphorylated level of the PI3K/AKT/FoxO3a pathway was decreased by dexamethasone treatment, resulting in elevated expression of Atrogin-1 and MuRF1. Interestingly, dexamethasone-induced muscle atrophy was remarkably reversed by trimetazidine, whereas inhibition of the PI3K/AKT pathway almost diminished the protective effects of trimetazidine. Our results are consistent with previous studies showing that trimetazidine exerts anti-atrophy effects in skeletal muscle [19, 30]. Indeed, the improvement of trimetazidine treatment on physical performance and muscle endurance has been demonstrated in patients with ischemic heart disease [31, 32]. Further clinical studies are needed to assess the efficacy and safety of trimetazidine treatment in patients with muscle dysfunction or sarcopenia.

Inflammation has been identified as the major mechanism for muscle atrophy [33]. Various stimuli can be sensed by pro-inflammatory Caspase-1 and lead to the cleavage of GSDMD which induces pyroptosis by forming membrane pores and increasing the secretion of proinflammatory factors such as IL-1β and IL-18 [34]. Recently, Ding et al. [13] showed that pyroptosis was activated in the gastrocnemius of cigarette smoke exposure-induced muscle atrophy mice model. Our present study demonstrated that dexamethasone treatment increased the cleavage and activation of pyroptosis-related genes Caspase-1 and GSDMD both in C2C12 myotubes and in mice. Inhibition of pyroptosis by silencing GSDMD significantly alleviated dexamethasone-induced atrophy in C2C12 myotubes. Although glucocorticoids are commonly used as anti-inflammatory agents, our results provide new insights into mechanisms of how sustained or high-dose use of glucocorticoids causes muscle pyroptosis and atrophy.

The activation of the NLRP3 inflammasome may be an important mechanism for glucocorticoid-induced myotube pyroptosis. The NLRP3 inflammasome cleaves pro-inflammatory IL-1β and IL-18 [12]. Emerging literature shows that NLRP3 inflammasome is activated in skeletal muscle in multiple muscle atrophy models [34–37]. Moreover, previous studies indicate that glucocorticoids can also modulate the assembly of the NLRP3 inflammasome [38, 39]. Consistently, dexamethasone treatment increased the expression of NLRP3 and Caspase-1 in C2C12 myotubes and mice. Knockdown of NLRP3 attenuated dexamethasone-induced muscle pyroptosis and atrophy, indicating that dexamethasone triggers myotube pyroptosis via activating the NLRP3 inflammasome. Taken together, these data suggest that NLRP3 may potentially be a therapeutic target for the treatment of skeletal muscle atrophy.

Another interesting finding of our study is that dexamethasone-induced pyroptosis was also attenuated by trimetazidine treatment. Conversely, activation of NLRP3 eliminated the protective effects of trimetazidine. Trimetazidine is a partial fatty acid oxidation inhibitor due to the suppression of long-chain 3 ketoacyl coenzyme A thiolase activity [40]. Recent studies have shown that trimetazidine possesses anti-inflammatory properties and plays cytoprotective roles in various tissues, including heart, brain, and renal tissues [41–43]. In the septic cardiac dysfunction model, trimetazidine attenuated LPS-induced cardiomyocyte pyroptosis by promoting neutrophils recruitment to the heart tissue [21]. Moreover, previous studies have demonstrated that mitochondria dysfunction precedes and activates muscle atrophy signaling in dexamethasone-induced muscle atrophy models [44, 45] and trimetazidine can target the mitochondria to improve mitochondrial function and prevent muscle atrophy under several wasting conditions [18, 30]. We now provide one mechanistic explanation for the therapeutic potential of trimetazidine in glucocorticoid-induced muscle atrophy in that it inhibits pyroptosis. Consistent with this, trimetazidine treatment decreases dexamethasone-induced upregulation of pyroptosis-related protein in skeletal muscle.

## Conclusions

**Fig. 7:**
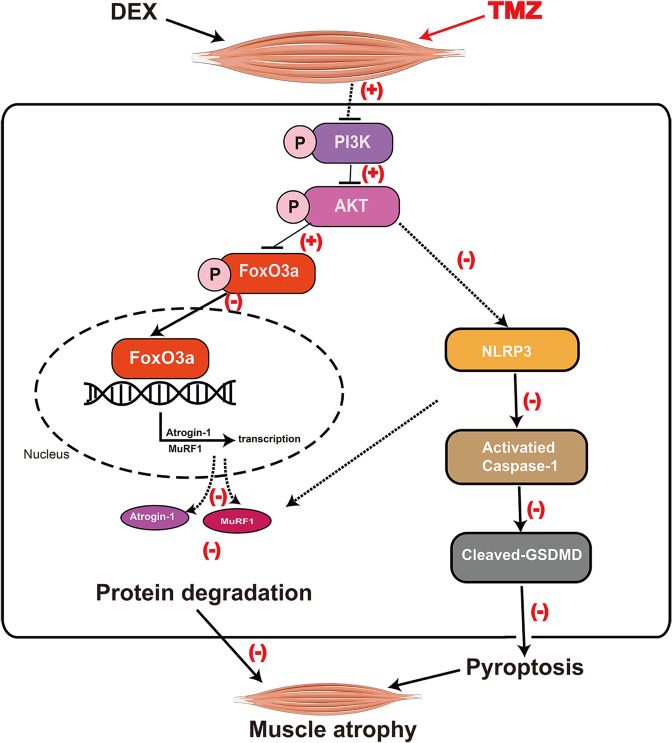
Schematic of the mechanism by which TMZ attenuates DEX-induced skeletal muscle atrophy. TMZ possesses a protective effect against DEX-induced skeletal muscle atrophy via promoting the phosphorylation of the PI3K/AKT pathway, which in turn inhibits FoxO3a-mediated transcriptional activation of Atrogin-1 and MuRF1 and NLRP3/GSDMD pathway-mediated pyroptosis. Black arrows represent the role of DEX, red plus (+), and minus signs (−) represent the role of TMZ. DEX dexamethasone, TMZ trimetazidine.

In summary, our data reveal a novel mechanism of dexamethasone-induced skeletal muscle atrophy. Dexamethasone induces muscle atrophy through suppressing of the PI3K/AKT pathway, which in turn not only promotes the dephosphorylation and nuclear translocation of FoxO3a but also activates NLRP3/Caspase-1/GSDMD pathway-mediated pyroptosis. Trimetazidine may alleviate dexamethasone-induced skeletal muscle atrophy partially via activating PI3K/AKT pathway and inhibiting NLRP3 (Fig. 7). Therefore, trimetazidine might be developed as a potential therapeutic agent for the treatment of dexamethasone-induced muscle atrophy.

## Supplementary information

Supplementary Figure Legends

Table S1

Table S2

Figure S1

Figure S2

Figure S3

Figure S4

## Data Availability

All data and materials used for this study are displayed or can be displayed upon request.
