# Supplementary material for: Trimetazidine attenuates dexamethasone-induced muscle atrophy via inhibiting NLRP3/GSDMD pathway-mediated pyroptosis
Source: Cell Death Discov. 2021 Sep 18;7:251. doi: 10.1038/s41420-021-00648-0 (PMC8449784; doi:10.1038/s41420-021-00648-0)
Supplement: Supplementary file 1 — Supplementary Figure Legends [file 41420_2021_648_MOESM1_ESM.docx]

**Supplementary Figure Legends**

**Figure S1. C2C12 myoblasts differentiate into mature myotubes.** D0, C2C12 myoblasts differentiated for 0 days. D4, C2C12 myoblasts differentiated for 4 days. Scale bar = 50μm.

**Figure S2. Relative fold change of C2C12 myotube diameter.** (A&B) C2C12 myotubes were treated with 10μM DEX combined with siNC or siRNA for 24 hours. C2C12 myotubes were transfected with siRNA targeting GSDMD (siGSDMD) or NLRP3 (siNLRP3). Diameter of C2C12 myotubes were analyzed. n=4, Data are shown as mean ± SEM. ***p＜0.01*，DEX+siNC or DEX *vs.* Ctrl. *##p＜0.01*，DEX+siRNA *vs.* DEX+siNC. (C) C2C12 myotubes treated with 10μM DEX for 24h and 150μM TMZ in the last 6h. Diameter of C2C12 myotubes were analyzed. n=4, Data are shown as mean ± SEM. ***p＜0.01*，DEX *vs.* Ctrl. *##p＜0.01*，DEX+TMZ *vs.* DEX. DEX, dexamethasone. TMZ, trimetazidine.

**Figure S3. Ratios of Gast, TA, Sol and Quad muscle to body weight.** n=8. Gast, gastrocnemius; TA, tibialis anterior muscle; sol, soleus muscle; Quad, quadriceps. Data are shown as mean ± SEM. **p*＜0.05，***p*＜0.01，DEX vs. Ctrl. #*p*＜0.05, ##*p*＜0.01，DEX+TMZ vs. DEX. DEX, dexamethasone. TMZ, trimetazidine.

**Figure S4. Myofiber cross sectional area of Gast.** Mice were intraperitoneally injected with 0.9% saline (Ctrl), DEX (25 mg/kg), TMZ (5 mg/kg), or DEX (25 mg/kg) +TMZ (5 mg/kg) for 10 days. Gast, gastrocnemius. n=4, Data are shown as mean ± SEM. **p＜0.01，DEX vs. Ctrl. ##p＜0.01，DEX+TMZ vs. DEX. DEX, dexamethasone. TMZ, trimetazidine.
