# Supplementary material for: Trimetazidine attenuates dexamethasone-induced muscle atrophy via inhibiting NLRP3/GSDMD pathway-mediated pyroptosis
Source: Cell Death Discov. 2021 Sep 18;7:251. doi: 10.1038/s41420-021-00648-0 (PMC8449784; doi:10.1038/s41420-021-00648-0)
Supplement: Supplementary file 2 — Table S1 [file 41420_2021_648_MOESM2_ESM.docx]

**Table S1. Antibodies for Western Blot**

| Antibody | Source | Vendor | Catalog No. | Dilution |
| --- | --- | --- | --- | --- |
| Fbx32(Atrogin-1) | Rabbit | abcam | ab168372 | 1:1500 |
| Caspaase1 | Rabbit | abcam | ab179515 | 1:1000 |
| IL-18 | Rabbit | abcam | ab71495 | 1:1000 |
| P-AKT(Ser473) | Rabbit | Cell Signaling Technology | 9271s | 1:1000 |
| AKT | Rabbit | Cell Signaling Technology | 9272s | 1:1000 |
| p-FoxO3a (Ser253) | Rabbit | Cell Signaling Technology | #9466 | 1:1000 |
| FoxO3a | Rabbit | Cell Signaling Technology | #2497 | 1:1000 |
| Gasdermin D (L60) | Rabbit | Cell Signaling Technology | #93709S | 1:1000 |
| Cleaved Gasdermin D (Asp276) | Rabbit | Cell Signaling Technology | 50928s | 1:1000 |
| NLRP3 (D4D8T) | Rabbit | Cell Signaling Technology | #15101S | 1:1000 |
| IL-1β (3A6) | Mouse | Cell Signaling Technology | #12242S | 1:1000 |
| PI3K(p85α) | Mouse | Proteintech | 60225-1-Ig | 1:10 000 |
| GAPDH | Mouse | Proteintech | 60004-1-Ig | 1:10 000 |
| MURF-1(TRIM63） | Goat | R&D Systems | AF5366-SP | 1ug/ml |
