# Supplementary material for: Trimetazidine attenuates dexamethasone-induced muscle atrophy via inhibiting NLRP3/GSDMD pathway-mediated pyroptosis
Source: Cell Death Discov. 2021 Sep 18;7:251. doi: 10.1038/s41420-021-00648-0 (PMC8449784; doi:10.1038/s41420-021-00648-0)
Supplement: Supplementary file 3 — Table S2 [file 41420_2021_648_MOESM3_ESM.docx]

**Table S2. Primer sets for Real-time PCR analyses**

| Gene | Forward primer (5′ to 3′) | Reverse Primer (5′ to 3′) |
| --- | --- | --- |
| GAPDH | AGGTCGGTGTGAACGGATTTG | TGTAGACCATGTAGTTGAGGTCA |
| Atrogin-1 | CAGCTTCGTGAGCGACCTC | GGCAGTCGAGAAGTCCAGTC |
| MuRF1 | GTGTGAGGTGCCTACTTGCTC | GCTCAGTCTTCTGTCCTTGGA |
| NLRP3 | TGTGAGAAGCAGGTTCTACTCT | GACTGTTGAGGTCCACACTCT |
| GSDMD | CCATCGGCCTTTGAGAAAGTG | ACACATGAATAACGGGGTTTCC |
| Caspase-1 | AGGCATGCCGTGGAGAGAAACAA | AGCCCCTGACAGGATGTCTCCA |
