# Supplementary figures and images for: Trimetazidine attenuates dexamethasone-induced muscle atrophy via inhibiting NLRP3/GSDMD pathway-mediated pyroptosis
Source: Cell Death Discov. 2021 Sep 18;7:251. doi: 10.1038/s41420-021-00648-0 (PMC8449784; doi:10.1038/s41420-021-00648-0)

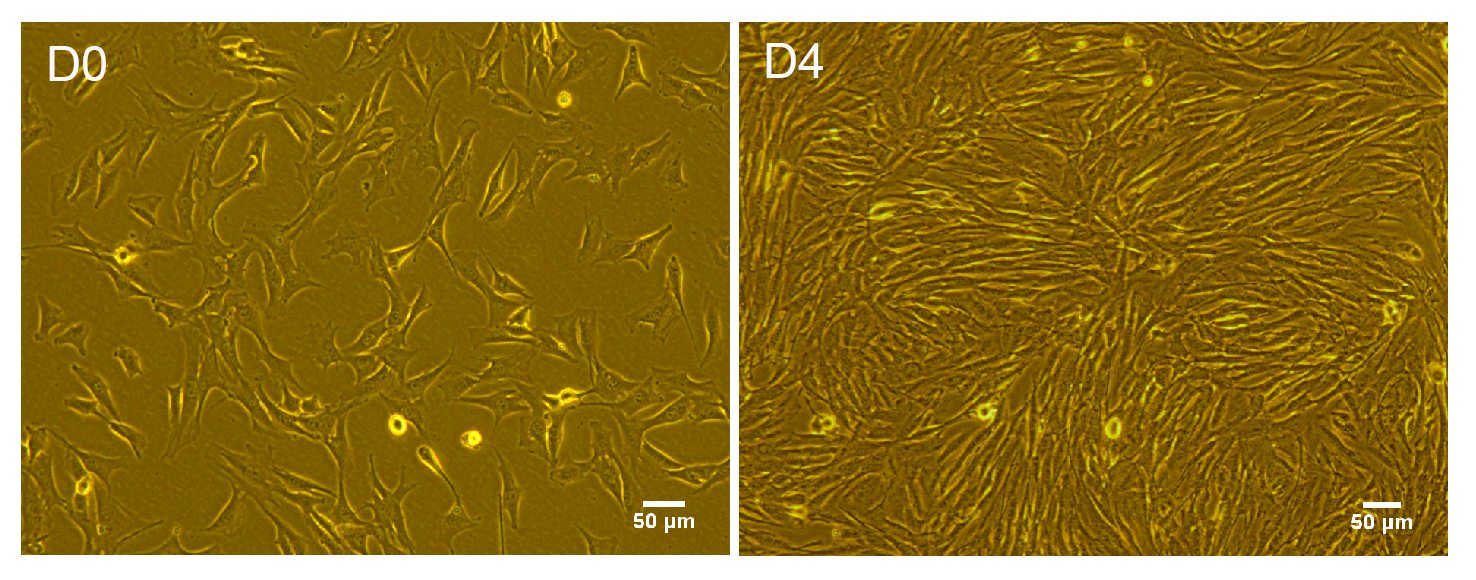

Supplement: Supplementary file 4 — Figure S1 [file 41420_2021_648_MOESM4_ESM.tif]

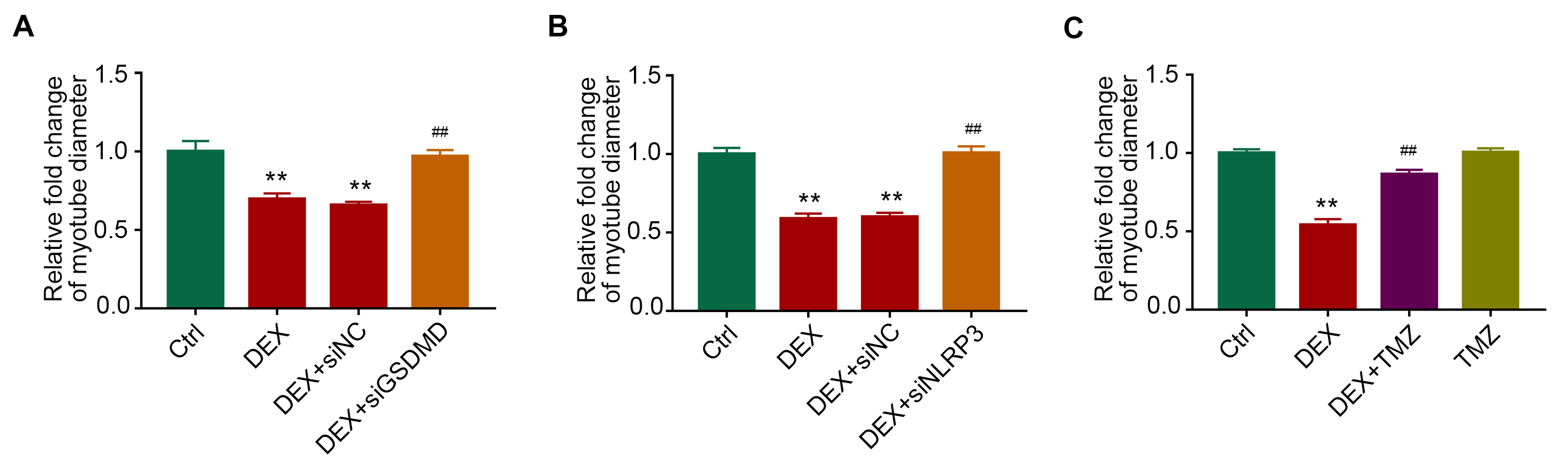

Supplement: Supplementary file 5 — Figure S2 [file 41420_2021_648_MOESM5_ESM.tif]

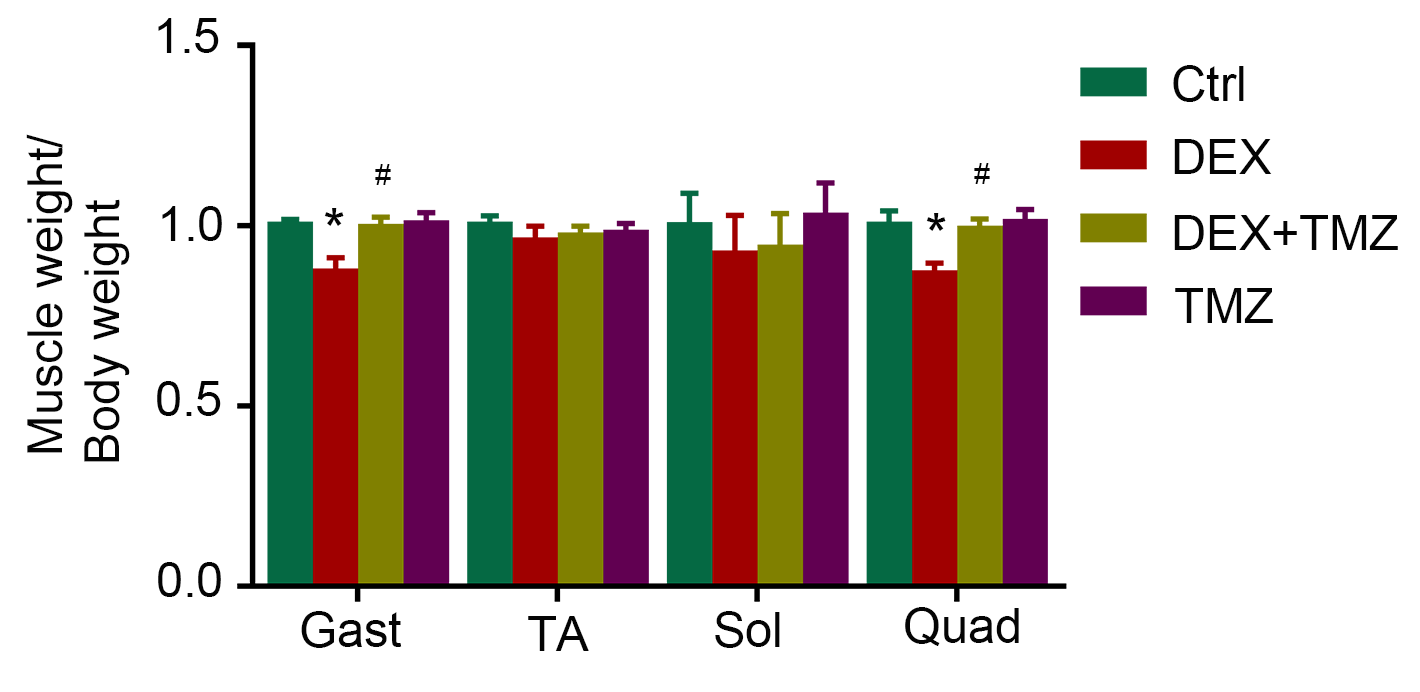

Supplement: Supplementary file 6 — Figure S3 [file 41420_2021_648_MOESM6_ESM.tif]

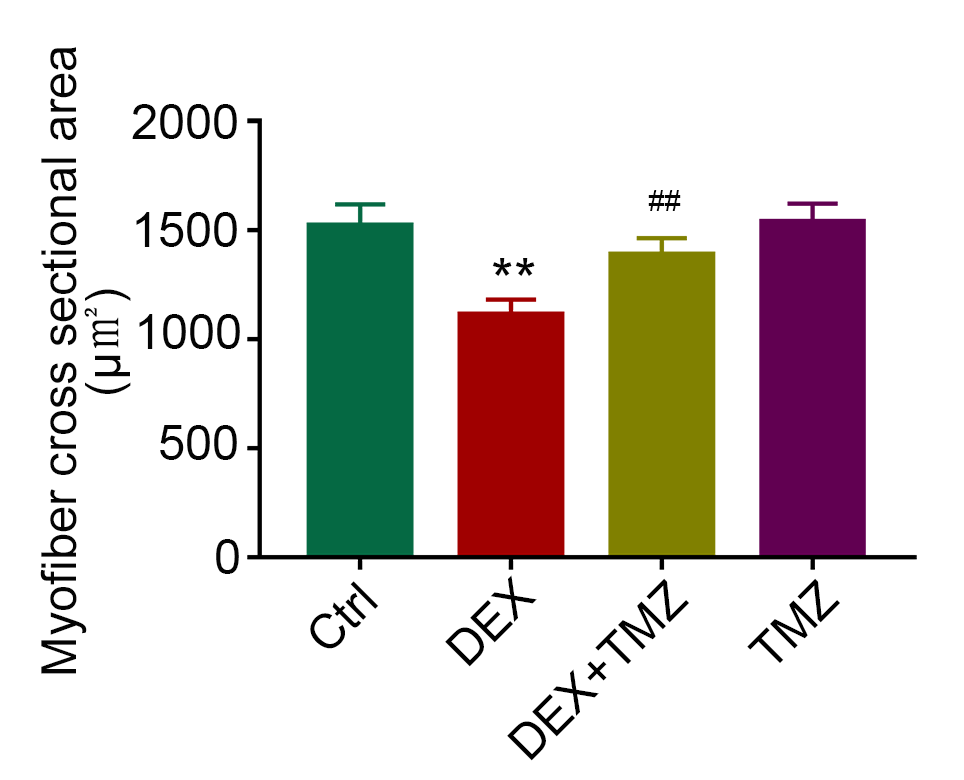

Supplement: Supplementary file 7 — Figure S4 [file 41420_2021_648_MOESM7_ESM.tif]
